# Adoption of radio-frequency identification to establish traceability in Taiwanese eel exported to the Japanese market

**DOI:** 10.1186/2193-1801-2-546

**Published:** 2013-10-17

**Authors:** Shu-Ching Jeng, Chun-Lung Wu, I-Da Yang

**Affiliations:** Former chief inspector of Inspection Center, Taiwan Eel and Shrimp Exporters’ Association, Kyoto, Japan; General manager, Homefull E-commerce LLC, 3F, No.162, Sec.3, Shin-Yi Road, Da-An Dist, Taipei, 106 Taiwan; Assistant professor, Department of Information Management, China University of Science and Technology, No. 245, Sec. 3, Academia Rd. Nangang Dist., Taipei City, 115 Taiwan

**Keywords:** eel, RFID, traceability, food safety

## Abstract

Eel culture and export to the Japanese market is an important industry in Taiwan; however, the average amount produced by each farm is small. Eels from different farms might be mixed before export, making it difficult to determine which farm is responsible for eels containing drug residues. Therefore, the Taiwanese government uses a two-stage procedure of inspection and accreditation for validating the use of good practice in aquaculture farming. Nevertheless, it is still difficult to trace any farm that has produced eels containing drug residues. Radio-frequency identification has the potential to establish traceability in eel products. Here we suggest that Japanese eel importers should insist on the use of radio-frequency identification by Taiwanese eel exporters to enable verification of the safety of eel products being exported to the Japanese market.

## Introduction

The aim of this article is to promote use of the RFID (radio frequency of identification) system in exportation of eel from Taiwan to Japan. Eel culture has been one of the most important aquaculture sectors in Taiwan and most of the products (about 90%) are exported to Japanese market. However, average production per farm is small and agencies are required to collect the eels for export. Eels also need to be graded at packing plants where there is a possibility of them getting mixed together. When an eel is found with drug residues, it is difficult to determine the farm where it was produced. Some farmers may take this as an opportunity to use drugs that are banned by the government. To solve the problem of use of prohibited drugs, the Taiwanese government take two responses. One is enforcement of two stages of inspection, and the other is accreditation of good aquaculture farms. Nevertheless, there are still some drawbacks of the two methods. Here, we suggest the adoption of the RFID system to establish traceability and prevent the use of drugs.

## The Taiwanese government’s efforts to overcome the problem of drug residues

To solve the problem of drug residues in eel exported to the Japanese market, the Taiwanese government uses two methods.

### Two stages of inspection

**Figure 1:**
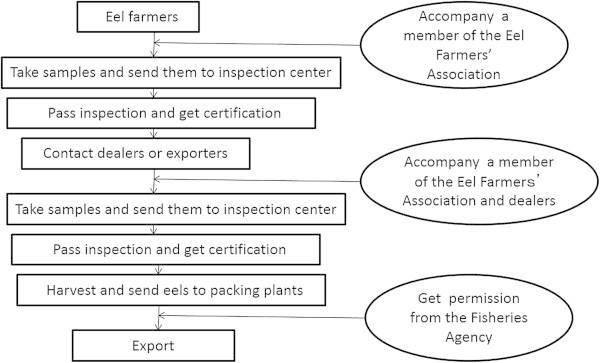
**Two-stage inspection procedure for live eel.**

To overcome the problem of drug residue traceability in eel farming, the Taiwanese government (i.e., The Fisheries Agency) implemented two stages of drugs inspection: the first before harvest and the second before export. To sell their eels, farmers are required to prove that these have been inspected and are free of drug residues. For this, an eel farmer accompanies a member of the Eel Farmers’ Association to the ponds that are to be harvested, and samples are taken and sent to the inspection center. Once certified, the farmer can contact dealers or exporters to sell the products. Before export, the exporter accompanies a member of the Eel Farmers’ Association to the ponds to be harvested, where further samples are taken and sent for inspection. After these two stages of inspection, the eel exporter needs to obtain permission from the Fisheries Agency to export their products to Japan. The entire process is shown in Figure 1.

However, this procedure has drawbacks for determining responsibility as there still exists a possibility that agencies mix the eels for export, owing to farms producing small amounts. Although the rules of this two-stage inspection procedure demands that each eel from every pond is inspected before export (second stage), agencies may not have all eels from every pond inspected to save on inspection fees. If an eel is found to contain drug residues, it is difficult to confirm the farm where it was produced because of the possibility of agencies or dealers having mixed all the eels together. Another worrying aspect is that Taiwan exports about 20,000 tons of eel to Japan annually; if every batch weighs about 2 tons, this equates roughly to 10,000 batches requiring inspection, and as every batch needs to be inspected twice, 20,000 inspections are needed. Assuming 200 working days per year, 100 batches require inspection every day. At present, sampling is done by the Eel Farmers’ Association, and this places a large burden on that body. Inevitably, mistakes occur.

**Figure 2:**
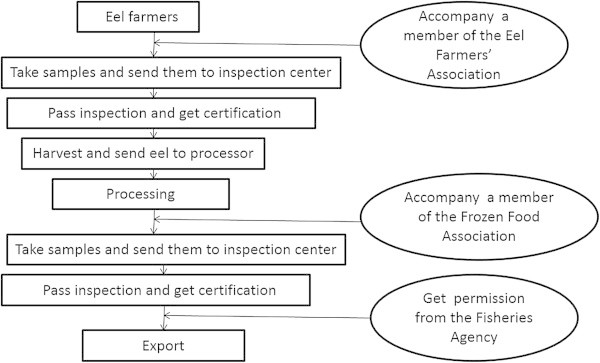
**Two-stage inspection procedure for processed eel.**

Next, we move on to the procedure for processing eels. After the first stage of inspection, eel from ponds that have been inspected can be sold to processing plants. After eel are processed, members of the Frozen Food Association are obliged to take samples of the processed eel and send these to inspection centers before export. After these two inspection stages, eel exporters need to obtain permission from the Fisheries Agency to export their eels to Japan. The entire process is shown in Figure 2. In this procedure, however, it is also difficult to determine the party responsible if processed eels are found to contain drug residues. Eel farmers may deny that those eel were sourced from their farms, which is difficult to prove.

### Accreditation of good aquaculture farms

To improve the quality of eel, in addition to the two-stage drug inspection process the Fisheries Agency is promoting Good Aquaculture Farms. By gaining accreditation, aquaculture farms can gain certification from the Taiwan Accreditation Foundation to demonstrate the quality of their farms and products. The Taiwan Accreditation Foundation has qualified experts who grant qualification to good aquaculture farms. Experts need to monitor the farm’s water quality, feed, operations, etc., to demonstrate a good aquaculture farm. However, more than 800 farms in Taiwan are small, making it difficult to allocate sufficient number of experts and time to monitor good aquaculture farms.

## Application of radio-frequency identification (RFID)

### The structure and fundamentals of RFID

**Figure 3:**
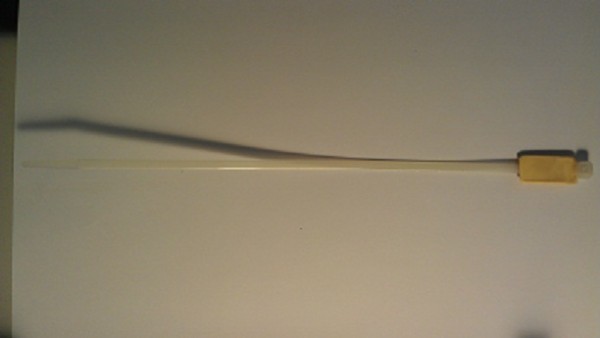
**An RFID chip.**

An RFID chip (Figure 3) is a type of electronic chip that uses a radio wave that can read and save 64–128 bytes of data. Apart from the fact that the storage capacity of an RFID chip is far superior to that of the common barcode (8 bytes), RFID is designed to input and read new information. Because RFID uses radio waves to read and save data, it is able to lock-down the user’s data; this is an advantage over barcodes, which are easy to duplicate.

Using RFID, the area, aqua-farmer, pond, and the production quantity of each farm can be recorded in detail and will not be duplicated, thereby establishing traceability when drug residues are found. However, when a radio chip is applied to two different materials, it cannot differentiate between those materials. To overcome this problem, the radio chip is combined with a waterproof material and an irreversible system. When the chip is attached to this material, providing the chip is not destroyed, the attached materials cannot be removed. By this means, it is easier to identify the source of the material.

### The suggested administration system for RFID

**Figure 4:**
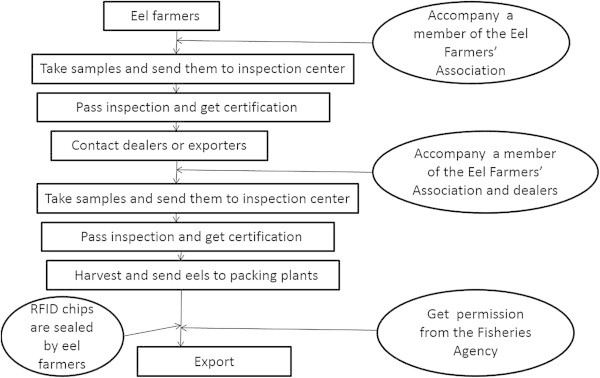
**The process of RFID administration for live eel.**

Until date, Taiwanese eel exporters have not used the RFID system to establish traceability of eels exported to Japan; therefore, we would like to inform and suggest it to them. We suggest that the Fisheries Agency should provide eel farmers with coded RFID chips in which the farmer’s details are recorded. After the two stages of inspection when the farmer decides to sell his eels, they need to confirm at the packing plant whether eels with only their RFID chips attached are packed together. RFID chips provided by the Fisheries Agency to each eel farmer are unique and cannot be transferred to other eel farmers, agencies, or any other body. However, if RFID chips are transferred, farmers would have to take responsibility for any eel with their RFID chips that were found to contain drug residues. The suggested process of administration is shown in Figure 4.

## Discussion

Awareness of food safety has enhanced the importance of traceability. Consumers question the origin and safety of the food they eat. Establishing traceability from farm to fork is a modern idea. A traceability system provides data that can backtrack the production chain from end user to producer, and even to the suppliers of the producer (Meuwissen et al. 2003). The purpose of traceability is to raise awareness of consumers that producers are being held accountable. At present, although it is difficult to track eel products from farm to fork, it can be established from farms in Taiwan to the Japanese market using RFID. In addition, postharvest processes represent another important aspect of food safety (Maita 2011). Although RFID is currently suitable for use only with live eel (its application is problematic in processed eel), this is an important initiative.

Following the occurrence of bovine spongiform encephalopathy cases in cattle, Japan established a traceability system for beef (Food Rink News 2003). Compared with cattle, eels are small and cannot be sold separately, making it difficult to establish traceability for every individual fish. However, it is possible to attach a traceable RFID chip to every packed unit of eel. Each packed unit of eel exported to the Japanese market weighs 20 kg, and every 20 kg package carries a traceable RFID chip. The cost of a piece of RFID chip is 20NT$. The price of eel is unstable and keeps changing. It can range from 200NT$ to 600NT$ per kg. The price of a packed unit (20 kg) can range from 4,000NT$ to 12,000NT$. If an RFID chip is attached to every packed unit, the cost will increase by about 0.5% when the price of a packed unit is 4,000NT$, and the cost will increase by about 0.17% when the price of a packed unit is 12,000NT$. For eel farmers and exporters, costs will slightly increase, but this extra cost is justified by the ease of traceability. RFID aside, eel farmers should keep records of their production process and the materials they use during production, as these are helpful in setting up a proper traceability system.

There are two types of aquaculture markets in Taiwan: demand-pull and supply-push (Jeng 2002). The development of a demand-pull market comes from consumer demand (Huisman 1986), and the eel market in Japan is of this type. Although, to date, Taiwanese eel exporters have not used the RFID system to establish traceability of eels exported to Japan, this system will be implemented as soon as importers in Japan ask for it to be used. For Japanese importers, since implementing RFID will increase costs while possibly not increasing profits, they might not consider it worthwhile to use RFID. Therefore, it is essential to give the public sufficient information on RFID as this system has the potential to improve the safety of eel produced for human consumption. It is to be hoped that the Japanese public will put pressure on importers and exporters to use RFID.

## Conclusion

In conclusion, RFID is strongly recommended to establish the traceability from farms in Taiwan to the Japanese market. The enforcement of the two stages of inspection and accreditation of good aquaculture farms can prevent use of drugs in aquaculture and improve the quality of products, but there are still some drawbacks and difficulties. Rather than depending only on these two ways, it is essential to ensure drug residue traceability in eel farming. We suggest using the RFID system. If eel farmers use RFID chips the cost will increase by about 0.17% to 0.5%, but it should not be a big burden for them. However, eel farmers will need to visit the packing plant to confirm that the correct RFID chips accompany their products. For eel farmers this may be problematic, but it can prove their desire to take responsibility and monitor the possible contamination that can occur when eels are being transferred from the farm to the packing plant. Although the use of RFID for this purpose will be a little more expensive for both Taiwanese exporters and Japanese importers, it will be beneficial for both of them when they understand the value of producing safe eels.
